# Endophthalmitis caused by gram-negative bacteria: etiologies, antibiotic susceptibilities, and treatment outcomes

**DOI:** 10.1186/s12348-026-00597-8

**Published:** 2026-05-23

**Authors:** Brandon Pham, Charles Zhang, Victor Sanchez, Harry W. Flynn, Darlene Miller

**Affiliations:** Department of Ophthalmology, Bascom Palmer Eye Institute, University of Miami Miller School of Medicine, 900 Northwest 17th Street, Miami, FL 33136 USA

**Keywords:** Gram negative endophthalmitis, Outcomes, Antibiotic susceptibilities

## Abstract

**Background:**

To report the etiologies, antibiotic susceptibilities, and treatment outcomes of patients with culture-proven endophthalmitis associated with gram-negative organisms.

**Methods:**

A single-center retrospective chart review of patients with positive vitreous cultures for gram-negative organisms who presented to a referral center from January 2014 to May 2025.

**Results:**

The study included 46 patients (mean age 69.5 years; 58.7% male). The most common isolates were *Pseudomonas aeruginosa* (19/46, 41.3%), *Haemophilus influenzae* (8/46, 17.4%), *Klebsiella pneumoniae* (3/46, 6.5%), *Morganella morganii* (3/46, 6.5% and *Serratia marcescens* (3/46, 6.5%). The most common etiologies were post-operative (20/46, 43.5%), followed by bleb-associated (9/46, 19.6%), corneal ulcer (6/46, 13.0%), trauma (5/46, 10.9%), endogenous (4/46, 8.7%), and suture-related (2/46, 4.3%). Most tested organisms were susceptible to amikacin (32/34, 94.1%), gentamicin (33/36, 91.7%), ceftazidime (36/37, 97.3%), tobramycin (33/35, 94.3%), and ciprofloxacin (34/37, 91.9%). Lower susceptibility rates were observed for trimethoprim-sulfamethoxazole (12/18, 66.7%) and ceftriaxone (13/15, 86.7%). All tested organisms were susceptible to meropenem (30/30, 100%). At the last follow-up, only 13.0% (6/46) of patients had a visual acuity of 20/800 or better, and 30.4% (14/46) of patients had no light perception. Enucleation or evisceration was performed in 28.3% of patients (13/46).

**Conclusion:**

In the current study, there were generally favorable susceptibilities to commonly used intravitreal antibiotics, but overall patients had poor visual outcomes regardless of specific causative organisms and etiologies. Despite appropriate treatment with intravitreal antibiotics and/or pars plana vitrectomy, enucleation and evisceration were common.

## Background

Endophthalmitis caused by gram-negative organisms is uncommon and can be associated with poor visual outcomes. In prior studies, 6% to 24% of all endophthalmitis cases have been reported to be caused by gram-negative bacteria [1–8]. Compared to gram-positive endophthalmitis, endophthalmitis caused by gram-negative organisms is generally associated with worse visual outcomes [9–11]. However, data pertaining to this uncommon condition remain limited, and there are considerable geographic variabilities in the epidemiology, microbiology, and antibiotic sensitivity profile of gram-negative endophthalmitis. The purpose of this report is to provide clinical experience regarding the most common gram-negative causative organisms, etiologies, antibiotic sensitivities, visual outcomes, and rates of evisceration/enucleation in these patients.

## Methods

This study is a single-center retrospective chart review of all patients with positive vitreous cultures for gram-negative organisms who presented to the Bascom Palmer Eye Institute from January 2014 to May 2025. Patients were initially diagnosed with endophthalmitis based on presence of vitritis either on exam or ultrasonography and confirmed with a positive vitreous culture. All patients with positive vitreous cultures of gram-negative isolates were included. Identification of organisms was performed using conventional microbiology procedures and the Vitek 2 system. Antimicrobial susceptibilities were evaluated using a combination of disc diffusion, E-tests, and Vitek 2. Patients with incomplete records (3) or no-follow up (5) were excluded. This study was approved by the University of Miami Institutional Review Board (IRB Approval #20120897) and conducted in accordance with the Declaration of Helsinki. Due to the retrospective nature of the study, a waiver of informed consent was approved for unidentified data acquisition. Written informed consent for publication of de-identified clinical images was collected for all patients through institutional consent forms. 

## Results

**Fig. 1:**
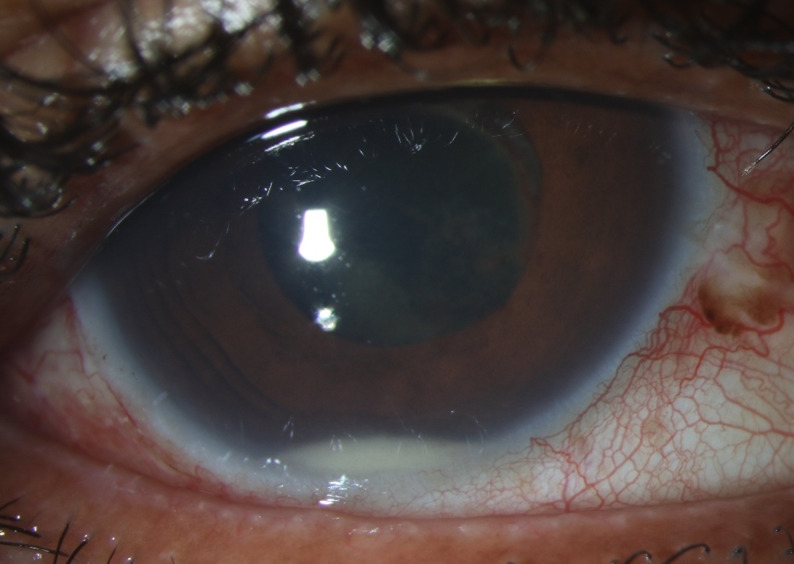
Slit lamp photo of right eye from a patient with post-operative endophthalmitis caused by *Pseudomonas aeruginosa.* A 51-year-old female initially presented to the emergency department one week after an uncomplicated cataract surgery in the right eye due to worsening vision. Visual acuity was 5/200 in the right eye, and she received a vitreous aspiration and injection of intravitreal vancomycin and ceftazidime. Cultures grew pan-sensitive *Pseudomonas aeruginosa.* Four days later, due to lack of clinical improvement, the patient underwent a pars plana vitrectomy with intravitreal injection of amikacin, ceftazidime, and dexamethasone with subsequent improvement in intraocular inflammation and resolution of her hypopyon. During the postoperative course, her best corrected visual acuity steadily improved to 20/200, which has been stable for one year after initial presentation

**Fig. 2:**
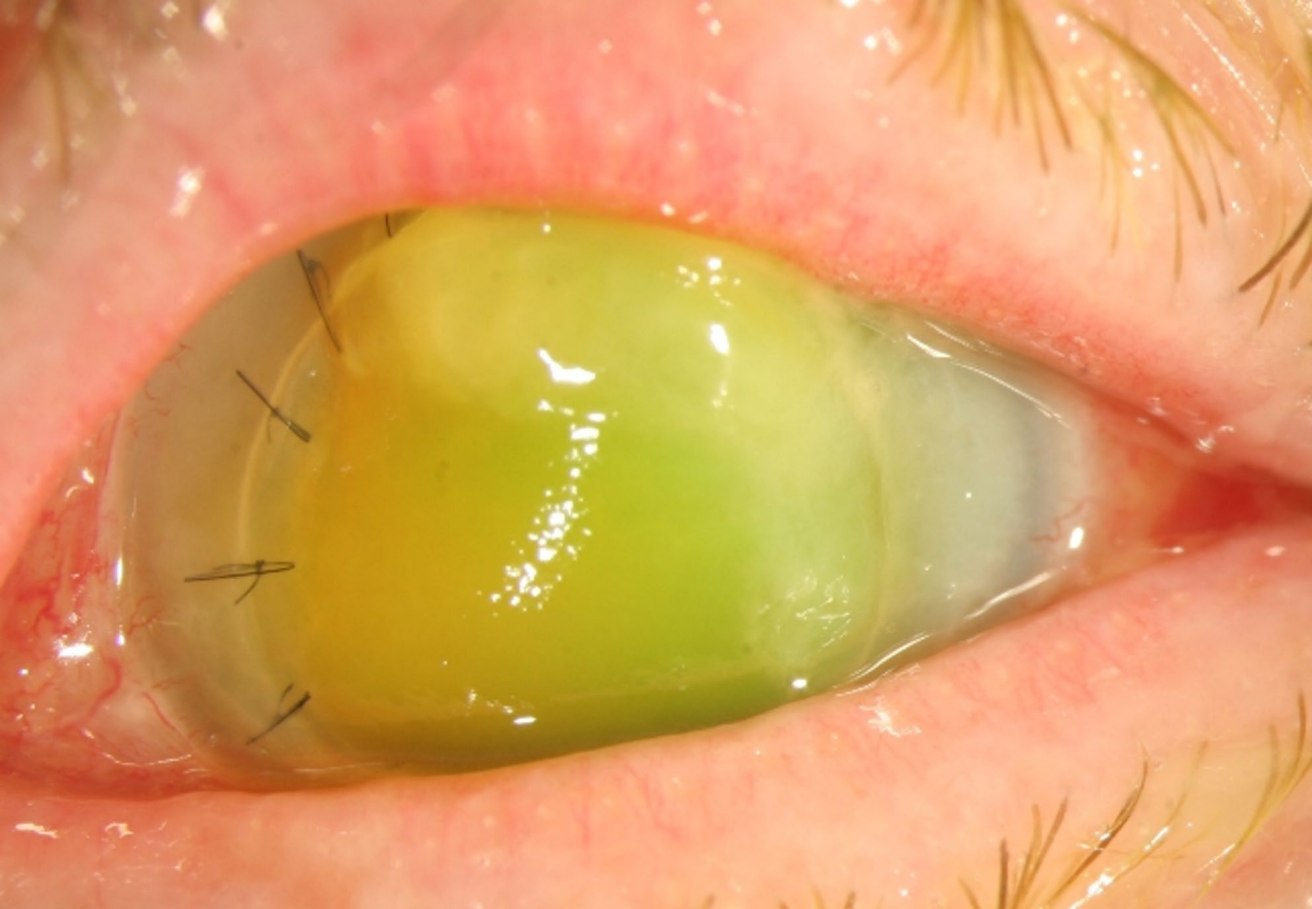
Slit lamp photo of left eye from a patient with endophthalmitis caused by *Morganella morganii* secondary to penetrating keratoplasty dehiscence. A 94-year-old male with a history of a penetrating keratoplasty in the left eye presented to the emergency room due to decreased vision and pain in the left eye. Examination revealed a dehisced penetrating keratoplasty, and visual acuity was no light perception (NLP). He received a vitreous aspiration and injection of intravitreal vancomycin and ceftazidime and was started on topical fortified vancomycin and tobramycin. Cultures grew *Morganella morganii*. Three days later, the patient continued to have NLP vision with significant pain. Enucleation was performed by the referring ophthalmologist

The study identified 46 patients (mean age 69.5 years; 58.7% male) with culture-confirmed gram-negative endophthalmitis. Systemic comorbidities included hypertension (65.2%), Type 2 Diabetes Mellitus (30.4%), Hyperlipidemia (23.9%), Rheumatic/autoimmune conditions (6.5%) Immunosuppression (6.5%), Cancer (4.3%), and Chronic Kidney Disease (4.3%). All cases consisted of unilateral endophthalmitis. The most common causative organism was *Pseudomonas aeruginosa* (19/46, 41.3%) (Fig. 1), followed by *Haemophilus influenzae* (8/46, 17.4%), *Klebsiella pneumoniae* (3/46, 6.5%), *Morganella morganii* (3/46, 6.5%) (Fig. 2), and *Serratia marcescens* (3/46, 6.5%). Less commonly isolated organisms included *Citrobacter koseri*, *Klebsiella aerogenes*, *Burkholderia cepacia*, *Escherichia coli*, *Pantoea*, *Achromobacter xylosoxidans*, *Rhizobium radiobacter*, *Neisseria meningitidis*, and *Moraxella*, each accounting for a single case (1/46, 2.2%) (Table 1). The most common clinical etiology was post-operative (20/46, 43.5%; Table 1), followed by bleb-associated (9/46, 19.6%), corneal ulcer (6/46, 13.0%), trauma (5/46, 10.9%), endogenous (4/46, 8.7%), and suture-related (2/46, 4.3%) (Table 1). All patients were initially treated with vitreous tap and empiric injections of vancomycin and ceftazidime. Final visual acuity of counting fingers or better was achieved in 23.9% (11/46) of patients.

**Table 1:** Gram-negative bacteria causing endophthalmitis in various etiologic categories

Etiology	Post-operative	Bleb/tube-related	Corneal ulcer	Trauma	Endogenous	Suture-related	Total (%)	Final visual acuity ≥ CF; No. (%)
*Pseudomonas aeruginosa*	10	1	6	2			**19 (41.3%)**	4/19 (21.1%)
*Haemophilus influenzae*	2	5				1	**8 (17.4%)**	3/8 (37.5%)
*Klebsiella pneumoniae*	1				2		**3 (6.5%)**	0/3 (0%)
*Morganella morganii*	2					1	**3 (6.5%)**	0/3 (0%)
*Serratia marcescens*	2	1					**3 (6.5%)**	1/3 (33.3%)
*Citrobacter koseri*	1				1		**2 (4.3%)**	0/2 (0%)
*Klebsiella aerogenes*				2			**2 (4.3%)**	0/2 (0%)
*Achromobacter xylosoxidans*	1						**1 (2.2%)**	1/1 (100%)
*Burkholderia cepacia*		1					**1 (2.2%)**	0/1 (0%)
*Escherichia coli*					1		**1 (2.2%)**	0/1 (0%)
*Moraxella*		1					**1 (2.2%)**	0/1 (0%)
*Pantoea*				1			**1 (2.2%)**	1/1 (100%)
*Rhizobium radiobacter*	1						**1 (2.2%)**	1/1 (100%)
Total	**20**	**9**	**6**	**5**	**4**	**2**	**46 (100%)**	**11/46 (23.9%)**

The susceptibilities of isolates in this study to selected antibiotics are summarized in Table 2. Most tested organisms were susceptible to amikacin (32/34, 94.1%), gentamicin (33/36, 91.7%), ceftazidime (36/37, 97.3%), tobramycin (33/35, 94.3%), and ciprofloxacin (34/37, 91.9%). Lower susceptibility rates were observed for trimethoprim-sulfamethoxazole (12/18, 66.7%) and ceftriaxone (13/15, 86.7%). All tested organisms were susceptible to meropenem (30/30, 100%).

**Table 2:** Gram-negative organism sensitivities, %

Organism	No. Tested	Amikacin	Gentamicin	Tobramycin	Ceftazidime	Ciprofloxacin	Meropenem	TMP/ SMX	Ceftriaxone
*Pseudomonas aeruginosa*	19	89.5 (17/19)	89.5 (17/19)	94.4 (17/18)	94.4 (17/18)	89.5 (17/19)	100 (17/17)	0 (0/2)	0 (0/2)
*Haemophilus influenzae*	7	NT	NT	NT	100 (3/3)	100 (4/4)	100 (1/1)	75 (3/4)	NT
*Klebsiella pneumoniae*	3	100 (3/3)	100 (3/3)	100 (3/3)	100 (3/3)	100 (2/2)	100 (2/2)	100 (2/2)	100 (2/2)
*Morganella morganii*	3	100 (2/2)	100 (3/3)	100 (3/3)*	100 (2/2)	50 (1/2)	100 (1/1)	50 (1/2)	100 (2/2)
*Serratia marcescens*	3	100 (3/3)	100 (3/3)	66 (2/3)	100 (3/3)	100 (3/3)	100 (3/3)	33 (1/3)	100 (3/3)
*Citrobacter koseri*	2	100 (2/2)	100 (2/2)	100 (2/2)	100 (2/2)	100 (2/2)	100 (2/2)	100 (1/1)	100 (2/2)
*Klebsiella aerogenes*	2	100 (2/2)	100 (2/2)	100 (2/2)	100 (2/2)	100 (2/2)	100 (1/1)	100 (1/1)	100 (1/1)
*Achromobacter xylosoxidans*	1	100 (1/1)*	0 (0/1)	100 (1/1)*	100 (1/1)	100 (1/1)	100 (1/1)	100 (1/1)	100 (1/1)
*Burkholderia cepacia*	1	NT	NT	NT	100 (1/1)	NT	100 (1/1)	100 (1/1)	NT
*Escherichia coli*	1	100 (1/1)	100 (1/1)	100 (1/1)	100 (1/1)	100 (1/1)	100 (1/1)	NT	100 (1/1)
*Moraxella*	1	100 (1/1)	100 (1/1)	100 (1/1)	100 (1/1)	100 (1/1)	NT	NT	100 (1/1)
*Pantoea*	1	NT	100 (1/1)	100 (1/1)	NT	NT	NT	NT	NT
Total	**44**	**94.1** **(32/34)**	**91.7** **(33/36)**	**94.3** **(33/35)**	**97.3** **(36/37)**	**91.9** **(34/37)**	**100** **(30/30)**	**66.7** **(12/18)**	**86.7** **(13/15)**

*NT*, not tested; *TMP/SMX*, trimethoprim/sulfamethoxazole. *Denotes intermediate sensitivity

The posttreatment visual acuities, rates of enucleation or evisceration, and change in visual acuity from baseline across different etiologies are summarized in Table 3. Visual acuities at last follow-up were no light perception (14/46, 30.4%), light perception (8/46, 17.4%), hand motion (5/46, 10.9%), counting fingers (5/46, 10.9%), and ≥ 20/800 (6/46, 13.0%). Evisceration or enucleation was ultimately performed in 13/46 (28.3%) of patients. In 12/13 (92.3%) of patients, eviscerations or enucleations were performed due to persistent pain in eyes with poor visual potential, while one was performed for cosmetic reasons per patient preference. From initial presentation to last follow-up, visual acuity improved in 14/46 (30.4%), was unchanged in 12/46 (26.0%), and worsened in 17/46 (37.0%) of patients.

**Table 3:** Posttreatment visual acuity for various etiologic categories

Etiologic Categories	Total No.	VA at last visit					Evisceration/ Enucleation	Change in VA from baseline		
		≥ 5/200	CF	HM	LP	NLP		Improved	No change	Worsened
Postoperative	20	3	4	1	7	5	6	6	8	6
Bleb/Tube-associated	9	1		3	2	3	3	3	2	4
Corneal ulcer	6		1	1	4		2	1	4	1
Trauma	5	1		2		2	1	1	1	3
Endogenous	4			1	1	2	2		1	3
Suture-related	2	1				1		1	1	
Total No (%)	**46**	**6** **(13.0%)**	**5** **(10.9%)**	**8** **(17.4%)**	**14** **(30.4%)**	**13** **(28.3%)**	**14** **(30.4%)**	**12** **(26.0%)**	**17** **(37.0%)**	**17 (37.0%)**

*CF*, counting fingers; *HM*, hand motion; *LP*, light perception; *NLP*, no light perception

Among patients who were treated with a pars plana vitrectomy (20/46, 43.5%), the causative organisms and visual outcomes are summarized in Table 4. Of these patients, the majority had a final visual acuity of worse than CF (14/20, 70.0%). Most of these patients (14/20, 70.0%) received a vitrectomy greater than three days after symptom onset.

**Table 4:** Organisms and visual outcomes among eyes that received pars plana vitrectomy (PPV)

Organism	Received PPV with final VA ≥ 5/200	Received PPV with final VA < 5/200
*Pseudomonas aeruginosa*	2	6
*Klebsiella pneumoniae*	0	1
*Klebsiella aerogenes*	0	2
*Haemophilus influenzae*	0	1
*Citrobacter koseri*	0	1
*Morganella morganii*	0	0
*Burkholderia cepacia*	0	0
*Escherichia coli*	0	0
*Pantoea*	0	0
*Serratia marcescens*	1	1
*Neisseria meningitidis*	1	0
*Rhizobium radiobacter*	1	0
Total	**6**	**14**

PPV, pars plana vitrectomy; VA, visual acuity

## Discussion

The current study reports updated clinical data on endophthalmitis caused by gram-negative bacteria. In the current series, the most common etiology and causative organism of gram-negative endophthalmitis were post-operative and *Pseudomonas aeruginosa*, respectively, which is consistent with prior reports [3]. Though less common in the United States than in Asia, *Klebsiella* species (Enterobacterales) were the second most common causative organisms of gram-negative endophthalmitis in this study [12]. This data confirms that gram-negative endophthalmitis generally has a poor visual prognosis, with most patients having a final visual acuity of 5/200 or worse. Moreover, despite early and appropriate treatment with intravitreal antibiotics and/or pars plana vitrectomy, 28.3% of patients in this series ultimately underwent evisceration or enucleation, which is similar to rates as high as 29.9% in other studies [13]. 

For patients with suspected infectious endophthalmitis, treatment usually consists of prompt vitreous aspirate and intravitreal injections of ceftazidime and vancomycin, which all patients in the current series received. The current study found that a majority of isolates were susceptible to ceftazidime which is in line with other past reports [14]; however, newer studies have also described cases of ceftazidime-resistant gram-negative endophthalmitis which required treatment with fluoroquinolones and imipenem [15]. Ciprofloxacin, in particular, may be an alternative to other fluoroquinolones and to ceftazidime or amikacin for gram-negative coverage [16], whereas imipenem may be best reserved for cases of multi-drug resistant cases [17]. The emergence of multi-drug resistant gram-negative organisms that are refractory to nearly all currently available antibiotics has presented a significant challenge to public health. There is an urgent need for the development of novel and alternative treatments for these organisms [18–21]. Data on the role of virulence factors, such toxins, biofilm, and necrosis factors, on outcomes are also needed. Due to the relatively uncommon nature of gram-negative endophthalmitis during this timeframe, the current study has several limitations, including its retrospective nature and a small sample size that was not adequate for statistical analyses. Certain data, such as those regarding sensitivities to specific antibiotics, were not available for all isolates. Importantly, in the current study treatment decisions did not follow a standardized protocol and were at the discretion of the individual physicians. Surgical intervention with pars plana vitrectomy was not associated with better visual acuities or reduced rates of enucleation and evisceration; however, this may be due to pars plana vitrectomy generally representing more severe cases with worse presenting visual acuities. Due to this reason as well as small sample size, the current study cannot make meaningful comparisons in visual prognosis across different etiologies, causative organisms or treatment modalities.

In the current study, there were generally favorable susceptibilities to commonly used intravitreal antibiotics including ceftazidime, but patients had poor visual outcomes regardless of specific causative organisms and etiologies. Despite prompt treatment with intravitreal antibiotics and/or pars plana vitrectomy, enucleation or evisceration were performed in 25% of eyes.

## Data Availability

The dataset generated from this study are available from the corresponding author upon reasonable request.
